# Dual‐Fuel Propelled Nanomotors with Two‐Stage Permeation for Deep Bacterial Infection in the Treatment of Pulpitis

**DOI:** 10.1002/advs.202305063

**Published:** 2023-12-03

**Authors:** Heping Wang, Xi Chen, Lulu Zhang, Ziwei Han, Jinxin Zheng, Yilin Qi, Weitao Zhao, Xihan Xu, Tianqi Li, Yutong Zhou, Pingping Bao, Xue Xue

**Affiliations:** ^1^ State Key Laboratory of Medicinal Chemical Biology College of Pharmacy Nankai University Haihe Education Park, 38 Tongyan Road Tianjin 300353 P. R. China; ^2^ Tianjin Key Laboratory of Oral and Maxillofacial Function Reconstruction Tianjin Stomatological Hospital The Affiliated Stomatological Hospital of Nankai University Tianjin 300041 P. R. China; ^3^ School of Medicine Nankai University Tianjin 300071 P. R. China; ^4^ Present address: Key Laboratory of Radiopharmacokinetics for Innovative Drugs Chinese Academy of Medical Sciences Tianjin Key Laboratory of Radiation Medicine and Molecular Nuclear Medicine Institute of Radiation Medicine Chinese Academy of Medical Sciences & Peking Union Medical College Tianjin 300192 P. R. China

**Keywords:** antibacterial activity, dual‐fuel propulsion, nanomotor, pulpitis therapy, two‐stage permeation

## Abstract

Bacterial infection‐induced inflammatory response could cause irreversible death of pulp tissue in the absence of timely and effective therapy. Given that, the narrow structure of root canal limits the therapeutic effects of passive diffusion‐drugs, considerable attention has been drawn to the development of nanomotors, which have high tissue penetration abilities but generally face the problem of insufficient fuel concentration. To address this drawback, dual‐fuel propelled nanomotors (DPNMs) by encapsulating L‐arginine (L‐Arg), calcium peroxide (CaO_2_) in metal‐organic framework is developed. Under pathological environment, L‐Arg could release nitric oxide (NO) by reacting with reactive oxygen species (ROS) to provide the driving force for movement. Remarkably, the depleted ROS could be supplemented through the reaction between CaO_2_ with acids abundant in the inflammatory microenvironment. Owing to high diffusivity, NO achieves further tissue penetration based on the first‐stage propulsion of nanomotors, thereby removing deep‐seated bacterial infection. Results indicate that the nanomotors effectively eliminate bacterial infection based on antibacterial activity of NO, thereby blocking inflammatory response and oxidative damage, forming reparative dentine layer to avoid further exposure and infection. Thus, this work provides a propagable strategy to overcome fuel shortage and facilitates the therapy of deep lesions.

## Graphical abstract

A new strategy for constructing dual‐fuel‐propelled nanomotors is developed to overcome the challenge of insufficient fuel concentration. The enhanced motion of nanomotors in pulp tissue promotes the deep delivery of nitric oxide (NO). The high penetration ability of the nanomotors and NO self‐diffusion collectively removes deep bacterial infection, thereby alleviating inflammatory response and forming a reparative dentine layer.

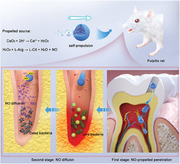

## 1 Introduction

Pulpitis is an inflammatory disease that occurs in the dental pulp and is caused by multiple (e.g., bacterial, physical, chemical, and immune) factors, among which bacterial infection is the most important one.^[^ ^1^ ^]^ During the early phase of infection, the inflammatory response serves as a protective role to eliminate invading bacteria and promotes tissue repair. However, when not timely treated, repeated and prolonged bacterial infections in deep caries can cause excessive immune cell infiltration and cytokines/chemokines release, thus stimulating severe inflammation and deep lesions formation in the pulp tissue.^[^ ^2^ ^]^ Persistent inflammation dramatically damages the proliferation and differentiation ability of dental pulp stem cells (DPSCs) and destroys the protective layer of dentin, ultimately leading to pulp function deficiency and tooth loss.^[^ ^3^ ^]^

Treatment options available for pulpitis with carious exposure range from conservative minimally invasive vital pulp treatments (VPT), including direct pulp capping (DPC), partial and complete pulpotomy, to the more invasive pulpectomy and root canal therapy (RCT).^[^ ^4^ ^]^ DPC and RCT are generally effective for reversible and irreversible pulpitis, respectively. With the development of biocompatible materials and an increasing understanding of healing processes triggered by carious dentin and pulp infections, cases traditionally deemed irreversible may be salvaged through VPT. Pulp capping materials play an important role in VPT.^[^ ^5^ ^]^ Ideal capping materials should also have specific properties such as good biocompatibility, antibacterial activity, reducing inflammatory response, promoting reparative dentin formation, and protecting pulp vitality. The commonly used pulp capping agents include calcium hydroxide, mineral trioxide aggregate (MTA), bioceramic materials, and calcium silicate materials.^[^ ^6^ ^]^ However, calcium hydroxide has an insufficient antibacterial effect, high solubility, and weak bonding strength, which makes it difficult to tightly seal the pulp and thereby causes leakage. MTA is also limited in clinical application due to its complex clinical operation, susceptibility to tooth discoloration, and potential cytotoxicity. Recently, iRoot BP Plus (abbreviated as iB Plus for marking), a novel bioceramic material, has an excellent pulp capping effect but is overly costly for broad clinical application.^[^ ^7^ ^]^ Moreover, the complex structure of the pulp renders techniques based on the passive diffusion of antibacterial agents ineffective.^[^ ^8^ ^]^ This easily leads to repeated bacterial infections in dental pulp tissue, exacerbating the development of pulpitis.

Unlike antibiotic therapy, nitric oxide (NO)‐based gas therapy does not pose the risk of resistance.^[^ ^9^ ^]^ The ultralow molecular weights and liposolubility endow NO with the ability to freely diffuse into biological membranes to kill bacteria by covalently binding to DNA, or proteins at appropriate concentrations.^[^ ^10^ ^]^ Nevertheless, the short half‐life of NO (several seconds) results in a short diffusion distance and poor tissue permeation ability.^[^ ^11^ ^]^ To improve the NO therapeutic effect on pulpitis, it is imperative to construct a novel delivery platform.

Diverse self‐propelling nanomotors have been developed to overcome the limitations imposed on micro‐/nanoparticle movement by disordered Brownian motion and low Reynolds number.^[^ ^12^ ^]^ It has been demonstrated that the self‐propulsion of nanomotors could enhance diffusion and penetration in organoids and tissue.^[^ ^13^ ^]^ Therefore, we intend to construct self‐propelled nanomotors with strong tissue permeability to deliver NO to deep lesions in the narrow pulp. NO could not only exert an antibacterial effect via self‐diffusion but also promote the movement of nanomotors for further penetration.^[^ ^14^ ^]^ For chemical‐fueled nanomotors, fuel concentration always increases the motion capability. Emerging studies have indicated that the movement performance of fuel‐propelled nanomotors is positively correlated with fuel concentration.^[^ ^15^ ^]^ The glucose oxidase/catalase cascade system is an attractive strategy to provide sufficient fuel in tissue with vigorous metabolism, such as tumors and the brain, which has been proven to supply enough driving force for nanomotor motion.^[^ ^16^ ^]^ In this case, the rapidly depleted endogenous H_2_O_2_ is regenerated by the oxidation of glucose in the presence of glucose oxidase, ensuring sustained O_2_ generation. However, as the glucose concentration in the pulp is insufficient for a reliable H_2_O_2_ supply, the above cascade system is not suitable for pulpitis therapy, which encourages us to construct a novel cascade system to achieve deep therapy in pulpitis.

**Scheme 1:**
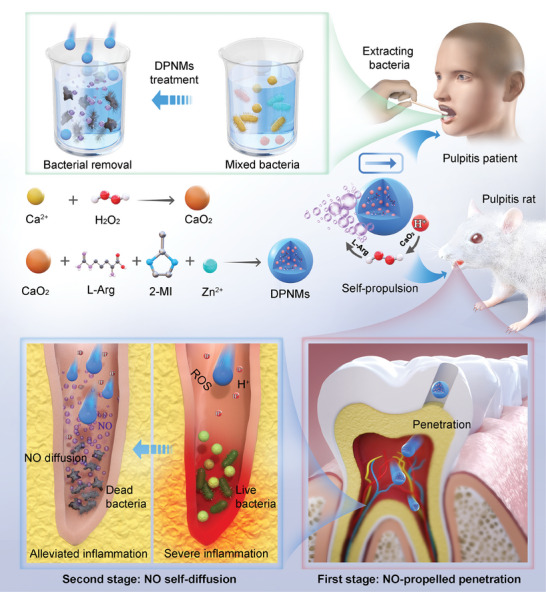
Schematic illustration of the preparation process, cascade reaction, and two‐stage permeation process of self‐propelled DPNMs in pulpitis therapy. Compared with traditional antibacterial drugs, our nanomotors could achieve two‐stage permeation on the basis of the NO‐propelled penetration and NO self‐diffusion. The strong permeability combined with the broad‐spectrum antibacterial property of NO not only kills the mixed bacteria extracted from pulpitis patients but also removes the deep‐seated bacterial infection and alleviates inflammation. The Ca^2+^ ions released from DPNMs further induce the osteogenic differentiation of dental pulp stem cells (DPSCs) and promote the formation of a protective dentin layer.

To address the need for the abovementioned alternatives, we herein constructed dual‐fuel propelled nanomotors (DPNMs) loaded with calcium peroxide (CaO_2_) and L‐arginine (L‐Arg), making use of the abundance of reactive oxygen species (ROS) and acidic molecules in the inflammatory microenvironment of the pulp.^[^ ^17^ ^]^ As shown in **Scheme** **1**, NO is produced by the reaction between L‐Arg and ROS, exerting antibacterial effects via self‐diffusion, and promoting nanomotor movement to enhance penetration. Uniquely, the consuming ROS could be supplemented through the reaction between CaO_2_ and H^+^. Results indicated that the released NO provides enough driving force for self‐propelled movement. Remarkably, compared to passive motion, fueled propulsion resulted in deeper penetration and thus facilitated the delivery of NO into the pulp interior. NO presented superior antibacterial activity against both Gram‐positive and Gram‐negative bacteria, substantially reducing the population of mixed bacteria extracted from patients with pulpitis. The synergy between nanomotor self‐propulsion and NO self‐diffusion enabled the efficient removal of deep bacterial infections, blocking the subsequent inflammatory response and oxidative stress while promoting the formation of a restorative dentin layer to prevent further exposure and infection of the pulp tissue. Overall, the implementation of DPNMs and the two‐stage permeation concept will provide theoretical guidance and a technical basis for intelligent nanomotors for disease therapy.

## 2 Results and Discussion

### 2.1 Characterization of DPNMs

**Figure 1:**
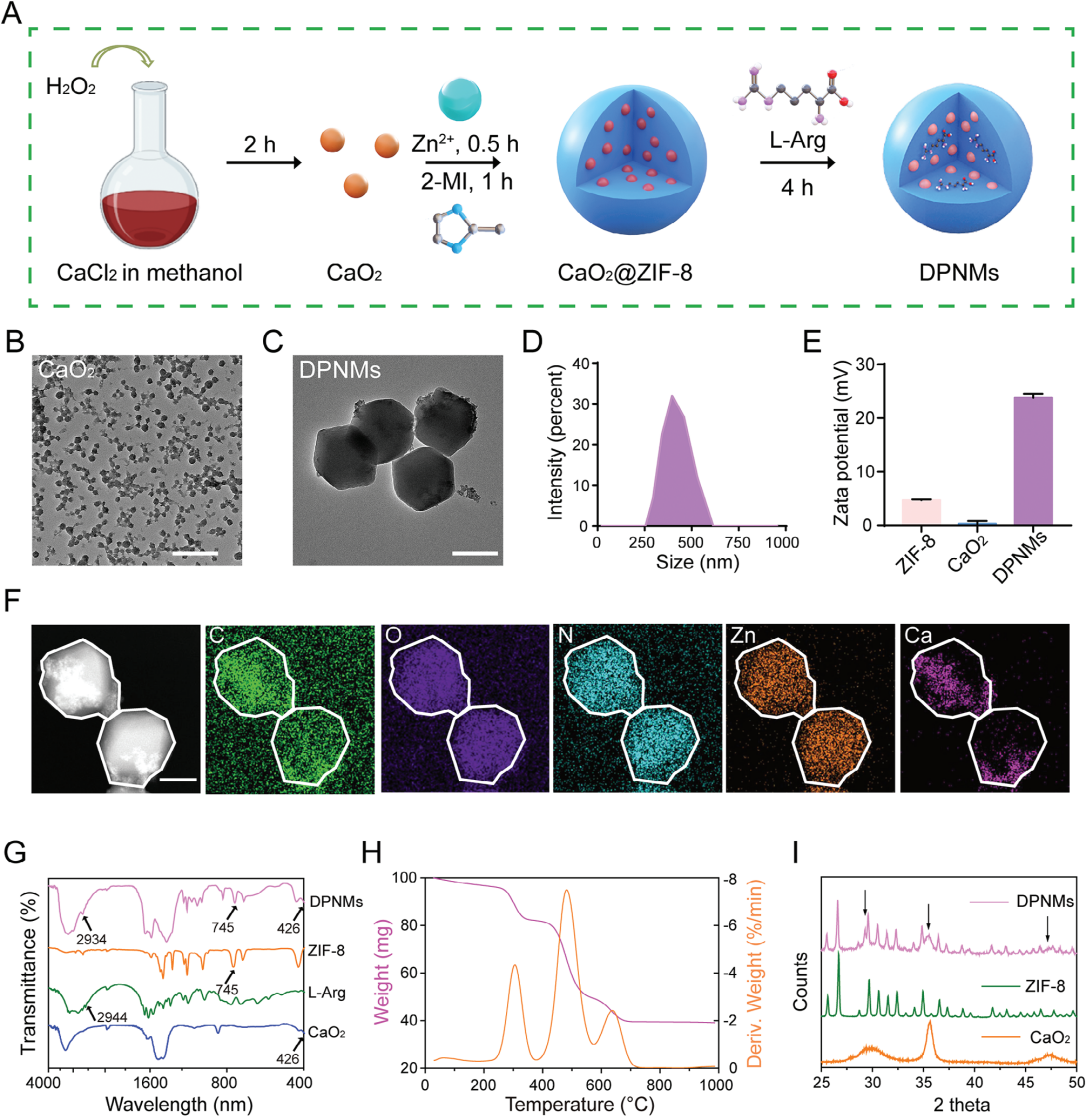
Synthesis and structural characterization of DPNMs. A) Schematic illustration of the synthesis of DPNMs. B) The TEM image of CaO_2_ naparticles. Scale bar, 200 nm. C) The TEM image of DPNMs. Scale bar, 200 nm. D) The size distribution of DPNMs detected by DLS. E) The zeta potentials of ZIF‐8, CaO_2_ nanoparticles, and DPNMs. F) The dark field image and corresponding elemental mappings of C, O, N, Zn, and Ca of DPNMs. Scale bar, 100 nm. G) The Fourier‐transform infrared (FTIR) spectrum of ZIF‐8, CaO_2_ nanoparticles, L‐Arg, and DPNMs. H) The thermogravimetric analysis of DPNMs. I) XRD pattern of the ZIF‐8, CaO_2_, and DPNMs.

In order to prepare DPNMs loaded with L‐Arg and CaO_2_, CaO_2_ nanoparticles were first synthesized by the reaction between H_2_O_2_ and calcium chloride (**Figure** **1**A). The transmission electron microscopic (TEM) image exhibited that CaO_2_ nanoparticles had a uniformly spherical morphology (Figure 1B). It was found from dynamic light scattering (DLS) results that the average size of CaO_2_ nanoparticles was about 20 nm (Figure S1, Supporting Information). After encapsulating CaO_2_ and L‐Arg in ZIF‐8 nanoparticles, DPNMs were completely prepared. Figure 1C exhibited that the size of DPNMs was about 290 nm. The average hydrodynamic diameter of DPNMs is approximately 361 nm (Figure 1D). Moreover, the polydispersity index (PDI) was 0.2, stating the good dispersity of DPNMs. The photographs also indicated that DPNMs could be well dispersed in aqueous solution (Figure S2, Supporting Information). Compared with ZIF‐8 and CaO_2_ nanoparticles, DPNMs possessed more positive surface potential, which may be due to the loading of L‐Arg (Figure 1E). Furthermore, elemental mappings of DPNMs stated the uniform distribution of C, O, N, and Zn elements throughout the entire structure (Figure 1F). The intense signal of Ca revealed the successful encapsulation of CaO_2_ nanoparticles. CaO_2_@ZIF‐8 and L‐Arg@ZIF‐8 nanoparticles with morphologies and sizes similar to those of the DPNMs were prepared as controls (Figure S3, Supporting Information). Owing to the encapsulation of L‐Arg, L‐Arg@ZIF‐8 showed more positive potential than CaO_2_@ZIF‐8.

The loading of L‐Arg and CaO_2_ was confirmed by Fourier transform infrared (FTIR) spectroscopy. The typical peak of ZIF‐8 at 745 cm^−1^ belonged to the vibration peaks of the imidazole ring skeleton (Figure 1G). The peak at 2944 cm^−1^ was ascribed to the characteristic absorption peak formed by the amino group of L‐Arg. The characteristic peak of CaO_2_ at 426 cm^−1^ was attributed to the peroxide. The corresponding characteristic peaks all emerge in DPNMs spectrum, further implying the loading of L‐Arg and CaO_2_ nanoparticles. The thermal stability of the DPNMs was determined via thermogravimetric analysis and its first derivatives. Results showed four‐stage degradation at approximately 66, 306, 480, and 636 °C, which represents the loss of water, L‐Arg, CaO_2_ and ZIF‐8 framework, respectively (Figure 1H). Meanwhile, the crystalline state of DPNMs was tested through X‐ray diffraction (XRD). XRD pattern showed DPNMs had a similar crystalline structure to ZIF‐8, but also showed obvious diffraction peaks at 29.4°, 35.6°, and 47.5° that belonged to CaO_2_ nanoparticles (Figure 1I). The loading content and encapsulation efficacy of L‐Arg were determined as 25.59 ± 1.77% and 2.05 ± 0.14%, respectively, while those of CaO_2_ were 7.67 ± 0.47% and 61.33 ± 3.75%, respectively. Taken together, these results demonstrated the successful preparation of nanomotors loaded with L‐Arg and CaO_2_.

### 2.2 Propulsion Sources and Motion Behavior of DPNMs

**Figure 2:**
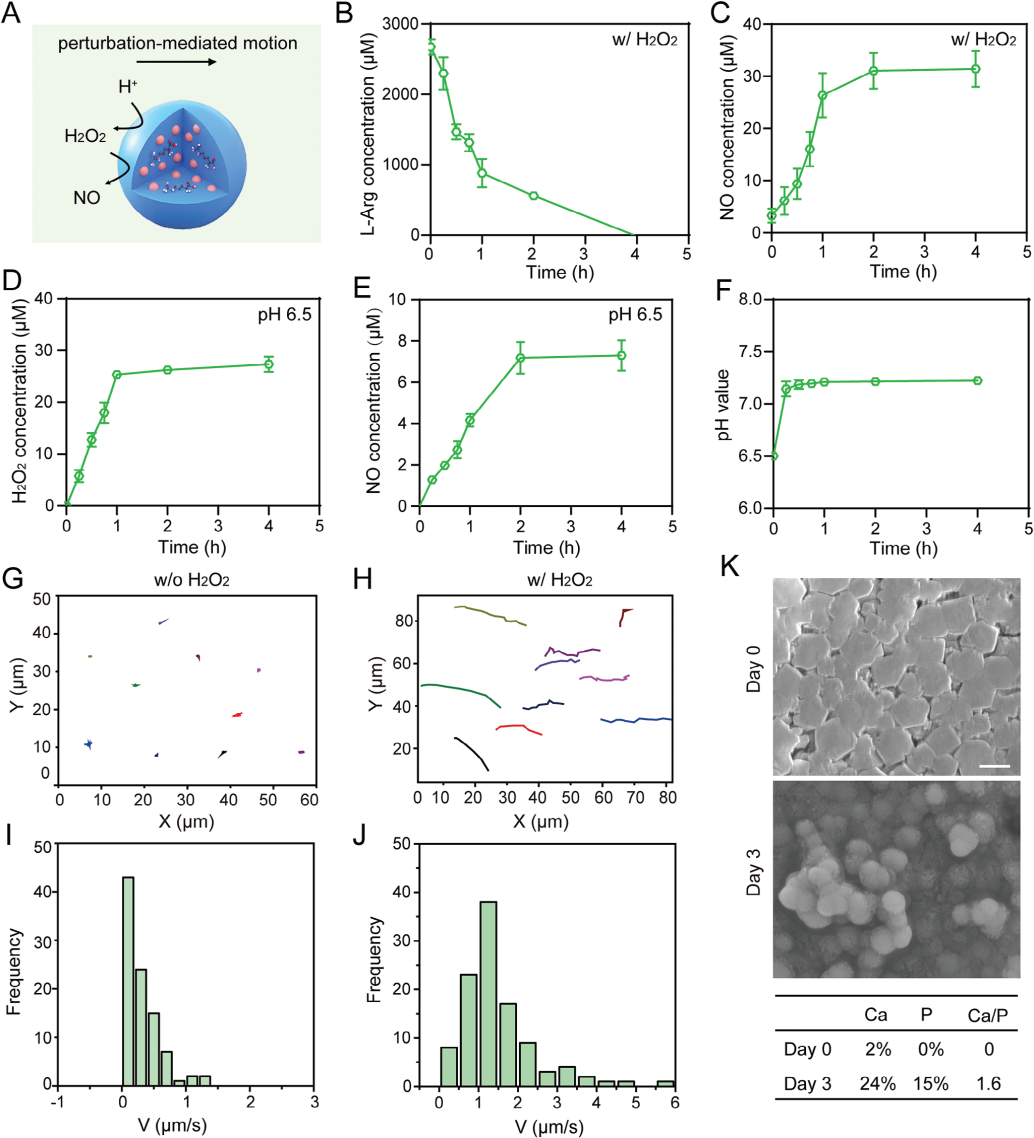
The NO release property from cascade reaction and motion behavior of DPNMs. A) Scheme illustration of the production of propulsion sources. B) The L‐Arg concentration of DPNMs in the presence of H_2_O_2_. C) The produced NO amount from DPNMs in the presence of H_2_O_2_ during the test time. D) The amount of H_2_O_2_ produced from DPNMs in the PBS solution at pH 6.5. E) The amount of NO produced from DPNMs in the PBS solution at pH 6.5. F) The change of pH when DPNMs are incubated with an acid solution. The trajectory of DPNMs without G) or with H) H_2_O_2_, and their corresponding statistical result I, J), *n* = 10. K) Representative SEM images of DPNMs before and after immersion in simulated body fluid (SBF) solution for 3 days. The table below shows the statistical calcium/phosphorus ratio. Scale bar, 200 nm.

Then, we elucidated whether the DPNMs could respond to ROS and acid to achieve enhanced movement. The cascade process is illustrated in **Figure** **2**A. We first tested the reaction between L‐Arg and H_2_O_2_, which provides the driving force for nanomotor movement. The concentration of L‐Arg was determined from the intensity of the absorption peak at 540 nm observed in the presence of 1‐naphthol and diacetyl as chromogenic agents. Figure 2B showed that the L‐Arg concentration rapidly decreased within 2 h, and seemed to disappear at 4 h. Meanwhile, the NO concentration was monitored by the nitrate reductase method. As a result, the trend of NO production was consistent with the consumption of L‐Arg, which indicated that NO was successfully generated by the reaction of L‐Arg and H_2_O_2_ (Figure 2C). This was also confirmed by the formed NO bubbles (Figure S4, Supporting Information). Next, the H_2_O_2_ production from CaO_2_ was evaluated. Given that H_2_O_2_ possesses a characteristic absorption peak at 240 nm,^[^ ^18^ ^]^ therefore, we ascertained the H_2_O_2_ production by UV spectrophotometry. In PBS solution at pH 6.5, the concentration of H_2_O_2_ rapidly increased within 1 h and subsequently reached equilibrium, which means that CaO_2_ in DPNMs could quickly generate H_2_O_2_ in the presence of H^+^ (Figure 2D). Notably, this is of great significance for overcoming insufficient fuel concentration, undoubtedly contributing to promoting the in vivo application of biomedical nanomotors. The generated H_2_O_2_ boosted the gradual accumulation of NO, verifying the proposed cascade process (Figure 2E). Moreover, the produced H_2_O_2_ was rapidly resumed by the loading L‐Arg, which will not cause additional damage to dental pulp tissue. The pH of the solution was further measured to assess the loss of H^+^. Results showed that the pH was changed to be slightly alkaline when incubating with DPNMs, which was beneficial to change the acidic microenvironment caused by bacterial metabolism and inflammatory response (Figure 2F). Collectively, the whole cascade processes of DPNMs under simulated pulpitis microenvironment can be described as follows:

(1) CaO2+2H+→Ca2++H2O2

(2) L−Arg+H2O2→NO+H2O+L−Cit

Considering that L‐Arg reacts with H_2_O_2_ to release NO, it is expected that the production of NO bubbles in the presence of H_2_O_2_ promotes active motion. Thus, we next investigated the movement behaviors of DPNMs upon H_2_O_2_ addition. The track trajectories of Cy5‐labelled DPNMs were recorded by a confocal laser scanning microscope. DPNMs exhibited significant self‐propelled motion behavior in dilute H_2_O_2_ solution, with significantly longer displacement than that in environments without H_2_O_2_ (Figure 2G,H; Movies S1 and S2, Supporting Information). Through statistical analysis of the movement displacement of ten nanoparticles, the average velocity of DPNMs in dilute H_2_O_2_ solution can reach up to 6 µm ^−1^s (Figure 2I,J). On the contrary, DPNMs have no obvious movement behavior in the environment without H_2_O_2_. To further investigate the self‐propelled movement behavior of the DPNMs over a long period, we developed a special device that is a two‐cell petri dish with a hole channel in the middle (Figure S5A, Supporting Information). After adding the DPNMs solution to one side, we removed a certain amount of liquid from the other side after a certain time. The content of the DPNMs in the dilute H_2_O_2_ solution was approximately 2.8 times that in the solution without H_2_O_2_ (Figure S5B, Supporting Information).

These data suggested that the H_2_O_2_‐caused NO release improved the movement distance and area of DPNMs. Moreover, to illustrate the role of CaO_2_ in the propelled process, we prepared L‐Arg@ZIF‐8 nanoparticles as a control group. The abovementioned dish was poured into a solution (pH 6.5) without H_2_O_2_. DPNMs or L‐Arg@ZIF‐8 nanoparticles were added into the solution according to the above‐mentioned method. Results indicated that DPNMs had higher movement ability than L‐Arg@ZIF‐8 nanoparticles, which may be due to the assistance of CaO_2_ (Figure S5C, Supporting Information). Although DPNMs do not have a typical Janus structure, they also exhibited enhanced motion with the assistance of H_2_O_2_ or H^+^. This may be due to the fact that geometric irregularities are inevitable in the process of preparing DPNMs.^[^ ^19^ ^]^ The asymmetric structure leads to an uneven distribution of the continuously generated NO bubbles around the nanomotor, which causes differential disturbing force, thus propelling the nanomotors.^[^ ^20^ ^]^ These results stated that the as‐prepared DPNMs could respond to the pulpitis microenvironment with severe oxidative stress and acidic substances to achieve self‐propelled movement, pointing out the great potential in treating deep bacterial infection.

Given that Ca^2+^ ions can form hydroxycarbonate apatite (HCA), which contributes to the occlusion of dentinal tubules and tooth remineralization,^[^ ^21^ ^]^ we investigated the formation of HCA after soaking the DPNMs in simulated body fluid (SBF) for three days. The representative SEM images exhibited that spherical nanoparticles appeared on the surface of DPNMs on the third day (Figure 2K). The elemental analysis showed the calcium/phosphorus ratio is 1.6, confirming the formation of HCA (Figure S6, Supporting Information). Afterward, the cytotoxicity of DPNMs was tested by MTT assay before in vivo administration. Results showed that DPNMs had negligible cytotoxicity at concentrations below 250 µg mL^−1^ (Figure S7, Supporting Information).

### 2.3 In Vitro Antibacterial Effect of DPNMs

**Figure 3:**
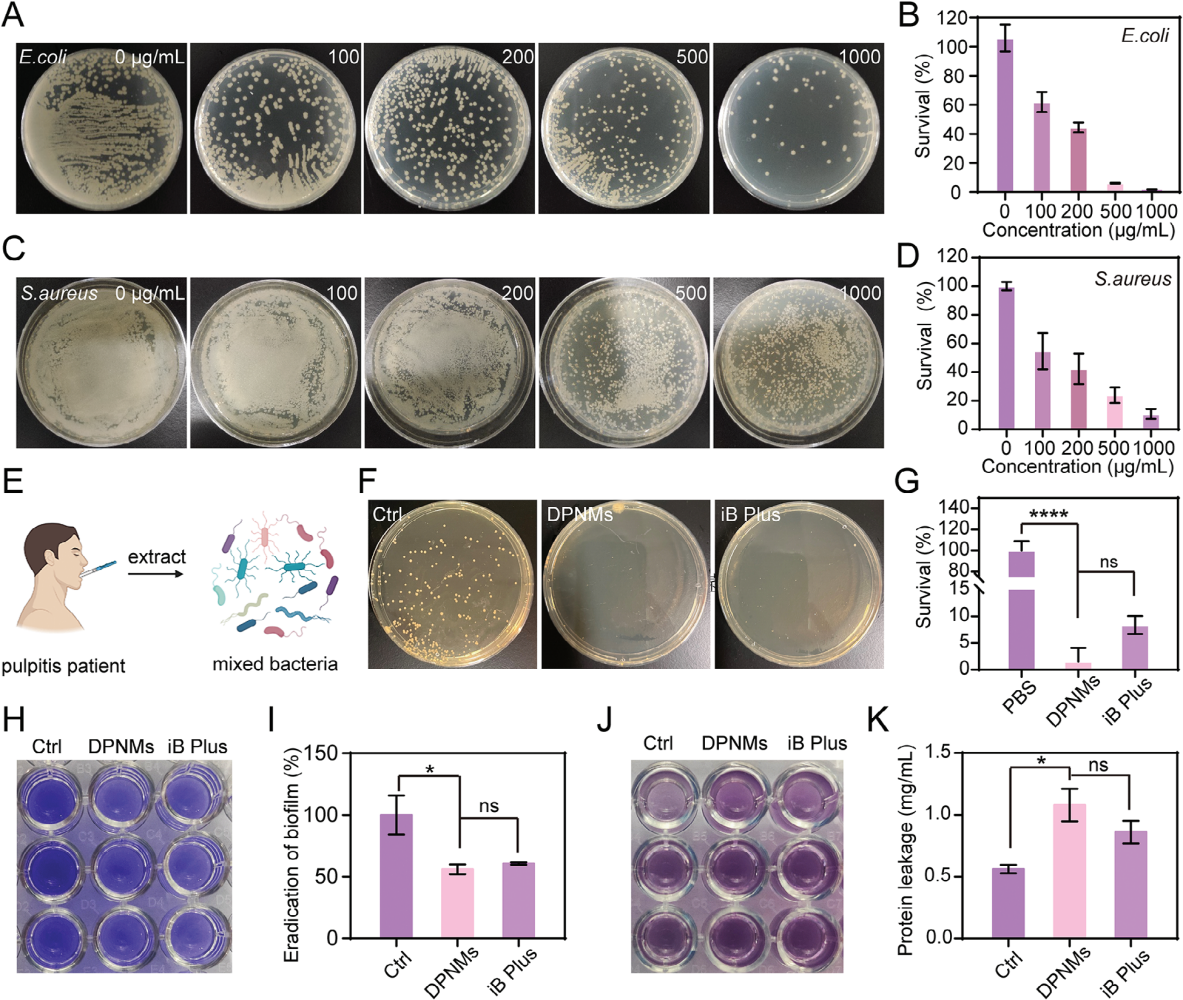
In vitro antibacterial effect of DPNMs. A) Representative colony formation and B) Survival rate of *E.coli* with the incubation of different concentrations of DPNMs (*n* = 3). C) Representative colony formation and D) Survival rate of *S.aureus* with the treatment of different concentrations of DPNMs (*n* = 3). E) Illustration of extracting bacteria in pulp cavity from pulpitis patients. F) Representative colony formation and G) Survival rate of mixed bacteria extracted from pulp cavity of pulpitis patients (*n* = 3). H) The photograph of crystal violet‐stained mixed bacteria biofilms. I) The quantitative analysis of relative biofilm biomass after different treatments (*n* = 3). J) The photograph of bicinchoninic acid‐measuring protein leakage and K) quantitative analysis (*n* = 3). G, I, K) Data are presented as mean ± SD, one‐way ANOVA, and Sidak's multiple comparison tests, *p < 0.05, and ****p < 0.0001 versus ctrl group, ns means no significance.

Mounting evidence has shown that bacterial infection is primarily responsible for the development of pulpitis.^[^ ^22^ ^]^ Therefore, we next assessed the antibacterial activity of NO released from DPNMs, revealing that decreased the plate counts of representative Gram‐negative (*Escherichia coli*) and Gram‐positive (*Staphylococcus aureus*) bacteria in a concentration‐dependent manner (**Figure** **3**A–D). Furthermore, DPNMs showed concentration‐dependent activity against bacteria. The antibacterial ability of NO originates from its high reactivity, which could form highly toxic peroxynitrite and dinitrogen trioxide with superoxide anion and H_2_O_2_.^[^ ^23^ ^]^ These molecules cause the nitrosation of thiols and amines on the cell surface and in the cell interior, thereby altering protein functions and inducing bacterial death.^[^ ^24^ ^]^ In addition, the highly toxic molecules also directly affect DNA, leading to DNA damage.^[^ ^25^ ^]^

However, the mixed bacteria in the dental pulp tissue includes a range of bacteria, rather than single *E. coli* and *S. aureus*.^[^ ^26^ ^]^ To comprehensively assess the antibacterial effect of DPNMs in pulpitis therapy, we extracted mixed bacteria of the pulp cavity from clinical pulpitis patients and further incubated that with our nanomotors (Figure 3E). As shown in Figure 3F,G, it is observed that the DPNMs treatment group and the iRoot BP Plus treatment group all have significant antibacterial effects, with an inhibitory rate of up to 90%. Compared to the clinically used iRoot BP Plus, the DPNMs exerted considerably stronger antibacterial effects on mixed bacteria, which was attributed to the broad‐spectrum antibacterial activity of NO.

Encouraged by the broad‐spectrum antibacterial activity, we further investigated the inhibitory effect of NO on biofilms that may be caused by residual bacteria in the pulp cavity. Mixed bacteria from pulpitis patients were cultured in 24 well plates to construct adherent biofilms. The change in biomass after different treatments was assessed by staining with crystal violet, which can bind to negatively charged molecules on the biofilm surface and polysaccharide components in the extracellular matrix. As shown in Figure 3H,I, DPNMs significantly decreased the biomass of biofilms in comparison with the control group. The eradicating effect of DPNMs is similar to that of iRoot BP Plus. The destruction of bacterial biofilm will result in the increase of the leaked protein. Therefore, we further detected the leakage protein in the upper culture medium with the bicinchonininc acid (BCA) protein assay kit. The protein leakage in the DPNMs and iRoot BP Plus groups exceeded that in the control group, which indicated that the DPNMs and iRoot BP Plus had a significant destructive effect on bacterial biofilms (Figure 3J,K). Collectively, the above results stated that DPNMs possessed broad‐spectrum antibacterial activity and exerted strong inhibitory effects on biofilms.

### 2.4 The Penetration Ability of DPNMs

**Figure 4:**
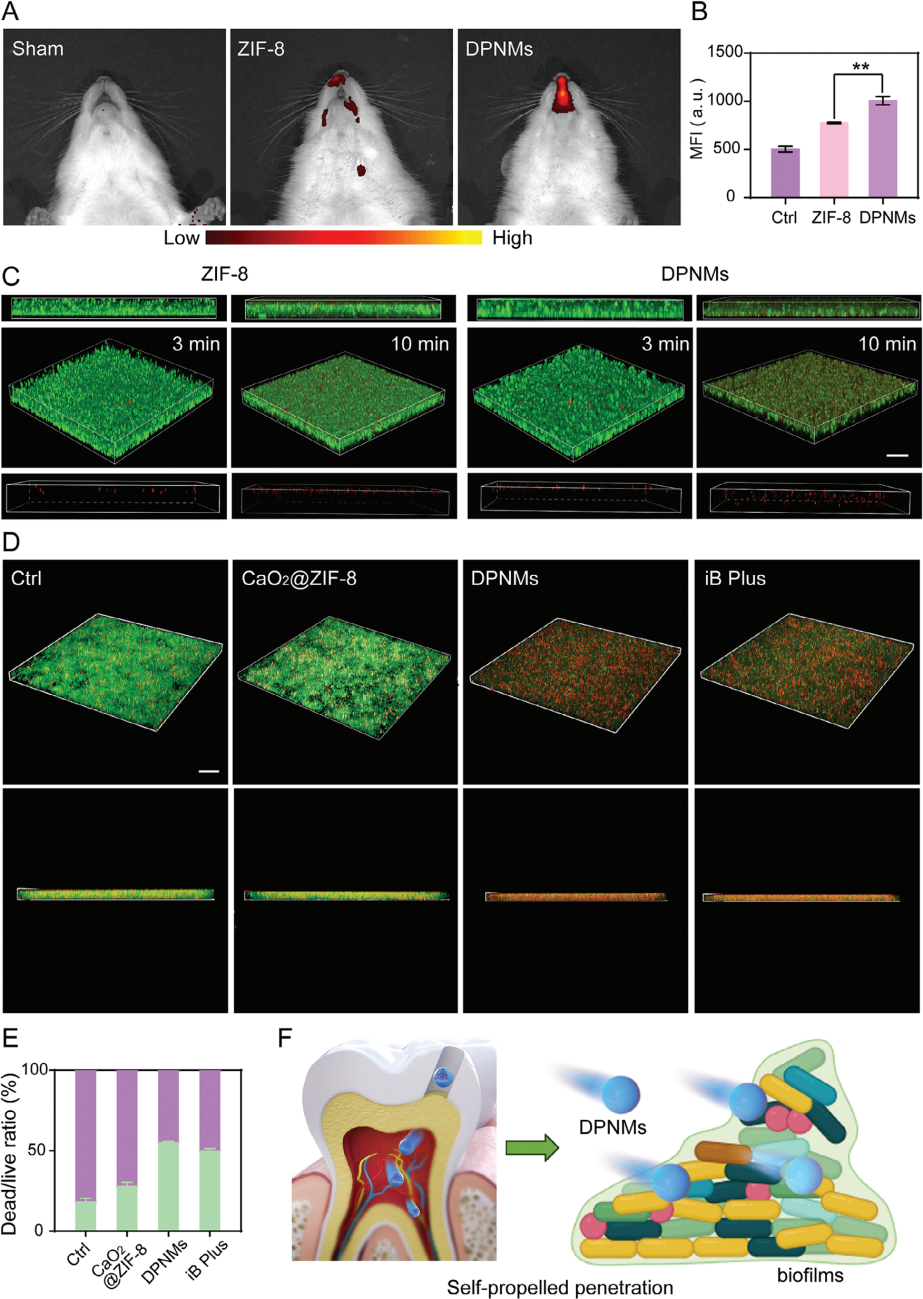
The evaluation of penetration ability of DPNMs. A) The IVIS imaging of Cy5‐labelled DPNMs in rats and B) the quantification of mean fluorescence intensity (MFI). Data are presented as mean ± SD (*n* = 3, one‐way ANOVA and Sidak's multiple comparison tests, **p < 0.01 versus ZIF‐8 group). C) 3D CLSM images and corresponding z‐stack images of mixed bacteria biofilms treated with Cy5‐labeled ZIF‐8 and DPNMs for 3 and 10 min. Scale bar: 30 µm. D) 3D CLSM images of mixed bacteria biofilms (green fluorescence: mixed bacteria biofilms stained with SYTO 9; red fluorescence: PI). Scale bar: 10 µm. E) The quantitative analysis of the dead/live ratio of bacteria. Data are presented as mean ± SD (*n* = 3). F) Illustration of NO‐propelled penetration in pulp tissue and biofilms.

The narrow spindle structure of the pulp cavity usually impedes the penetration of passive nanoparticles, complicating the treatment of bacterial infection at the bottom of the pulp. We next investigated the penetration ability of DPNMs. Cy5 was loaded into DPNMs to track the permeation in the pulpitis model. The IVIS imaging exhibited that self‐propelled DPNMs penetrated into the pulp cavity 4 h post‐treatment while passive ZIF‐8 nanoparticles failed to enter into the pulp cavity and therefore led to discrete fluorescence distribution due to effusion (**Figure** **4**A). Though nanoparticles with the same fluorescence were administrated, the DPNMs‐treated group showed stronger fluorescence intensity than the ZIF‐8‐treated group owing to enhanced retention by penetration (Figure 4B). The biofilm formed by residual bacteria in the pulp cavity usually induces the development of irreversible pulpitis.^[^ ^27^ ^]^ Thereupon, we next estimated the ability of the nanomotors to permeate the biofilms produced by culturing mixed bacteria from patients with pulpitis. Figure 4C showed that DPNMs emerged in a deeper position than ZIF‐8 during the same time, stating that the self‐propulsion of DPNMs promotes the deep penetration in the biofilms. Then, we investigated the antibiofilm capacity of DPNMs by live/dead staining. As shown in Figure 4D,E, DPNMs resulted in bacterial death from top to bottom, which achieved a similar antibacterial effect to iRoot BP Plus. The high antibiofilm capacity may be ascribed to the permeability of nanomotors and the diffusibility of NO with broad‐spectrum antibacterial activity. Reversely, CaO_2_@ZIF‐8 nanoparticles caused less bacterial death due to the lack of NO release. As expected, the released NO has the ability to boost the first‐stage permeation of DPNMs in pulp tissue and further achieve deep penetration in the biofilms (Figure 4F).

### 2.5 The Antibacterial and Anti‐Inflammatory Effect of DPNMs in Pulpitis Model

**Figure 5:**
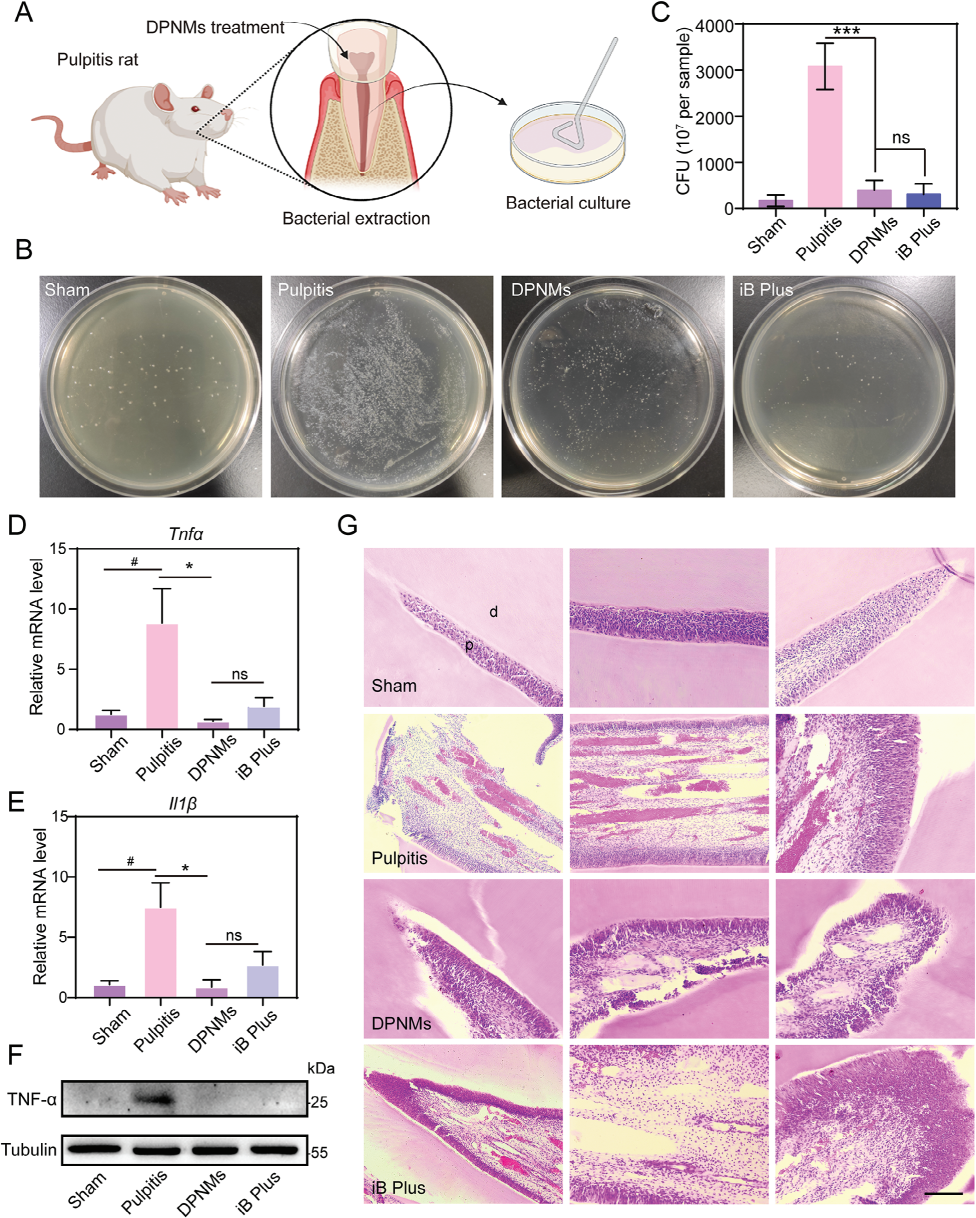
The antibacterial and anti‐inflammatory effect of DPNMs therapy in pulpitis model. A) Illustration of extracting bacteria from DPNMs‐treated rats. B) Representative colony formation and C) survival rate of mixed bacteria from rats after the different treatments. D, E) RT‐qPCR quantitative analysis of (D) *Tnfα*, (E) *Il1β* levels in pulp tissue with different treatment (*n* = 3). (F) Expression levels of TNF‐α in different groups by western blot (*n* = 3). G) H&E staining of pulp tissue with different treatments. Scale bar, 200 µm. (C, D, E) Data are presented as mean ± SD (one‐way ANOVA and Sidak's multiple comparison tests, ^#^ *p* < 0.05 versus sham group, and *p < 0.05, ***p < 0.001 versus pulpitis group, ns means no significance).

In view of the high penetration ability of the DPNMs, we then explored their capacity to reduce bacterial infection in a rat pulpitis model established by exposing the pulp tissue through the destruction of the enamel and dentin layers of the mandibular anterior teeth for 6 h.^[^ ^28^ ^]^ 28 days post‐administration, we extracted bacteria from dental pulp tissue and evaluated the antibacterial effect using the traditional plate count method (**Figure** **5**A). As shown in Figure 5B,C, the CFU count of the DPNMs‐treated group is significantly decreased in comparison with pulpitis rat, revealing outstanding antibacterial effects. The antibacterial ability of DPNMs is similar to iRoot BP Plus, which implies the practicability and potential in clinical application.

Such advances encouraged us to think about whether DPNMs could alleviate bacterial infection‐induced inflammatory response. Hence, we measured the levels of cytokines in pulp tissue and quantified proinflammatory factors using real‐time fluorescent quantitative polymerase chain reaction analysis. As shown in Figure 5D,E, pulpitis rat was accompanied by significant inflammation, increasing the level of proinflammatory factors (*Tnfα*, *Il1β*). However, this was reversed by DPNMs treatment and iRoot BP Plus. Notably, DPNMs showed a comparably anti‐inflammatory effect to iRoot BP Plus. This conclusion was further affirmed through western blotting (WB) analysis (Figure 5F; Figure S8, Supporting Information). WB results and quantitative data exhibited the excellent anti‐inflammatory performance of DPNMs from protein levels. The anti‐inflammatory property of DPNMs may originate from the significant inhibition of bacterial growth, which fundamentally blocks the initiation of the inflammatory cascade process.

Furthermore, we tested the tissue injury through hematoxylin and eosin (H&E) staining. In the sham group, no signs of pathology, necrosis, or significant calcification were observed, and the pulp tissue was normal without congestion (Figure 5G). After long‐term exposure to the oral cavity, necrotic areas were observed in the dental pulp tissue, which was accompanied by a significant increase of inflammatory cells, and extensive hemorrhagic necrosis. H&E staining showed that the congestion of the medullary cavity in the DPNMs group was significantly improved, and inflammatory cell infiltration was significantly reduced. The iRoot BP Plus group also significantly improved the congestion of the medullary cavity, while there was no significant improvement in vascular dilation.

### 2.6 DPNMs Induced Osteogenic Differentiation Potential of DPSCs

DPSCs have high proliferation, self‐renewal, and multi‐directional differentiation potential.^[^ ^29^ ^]^ DPSCs could differentiate into odontoblast and therefore form restorative dentin, keeping dental pulp tissue away from the interference of external stimuli. It is exceedingly necessary and imperative for pulpitis therapy. Emerging studies have reported that calcium ions in pulp capping agents are easy to induce the formation of HCA, which contributes to dentin tubules occlusion and teeth remineralization, and promotes the migration, proliferation, and differentiation of DPSCs, further boosting osteogenic differentiation to form reparative dentin layer.^[^ ^30^ ^]^ Considering that the DPNMs could form HCA in the SBF solution (Figure 2K), we next evaluated their ability to induce the differentiation of DPSCs and promote the formation of restorative dentin tissue.

**Figure 6:**
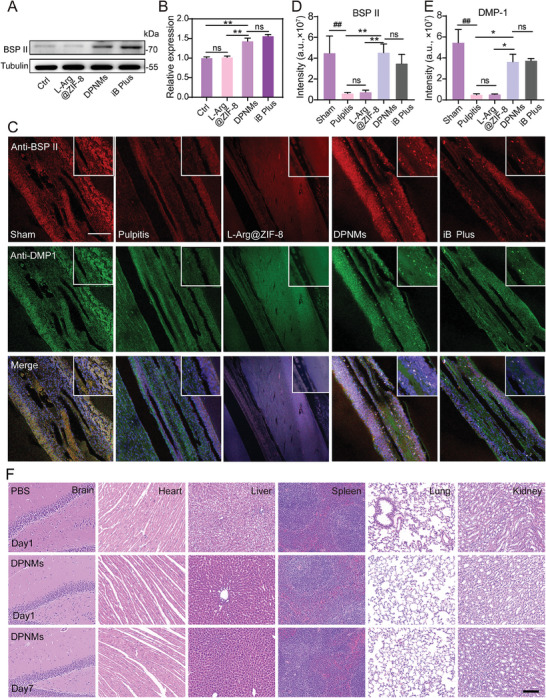
The osteogenic differentiation potential of DPSCs. A) Expression levels of BSP II in different groups by western blot. B) The quantization of BSP II expression by ImageJ software (*n* = 3). C) Immunofluorescence co‐staining of BSP II (red) and DMP1 (green) by CLSM in different groups. Scale bar = 100 µm. D, E) Quantitative analysis of the MFI of BSP II (D) and DMP1 (E) in immunofluorescence co‐staining results (*n* = 3). F) Representative images of H&E staining in the brain, heart, liver, spleen, lung, and kidney from WT mice with different treatments. Scale bar = 100 µm. (B, C, D) Data are presented as mean ± SD (one‐way ANOVA and Sidak's multiple comparison tests, ^##^ *p* < 0.01 versus sham group, and ^*^ *p* < 0.05, ^**^ *p* < 0.01 versus pulpitis group or DPNMs group, ns means no significance).

DPSCs were incubated with the DPNMs for testing differentiation, which was characterized by the levels of bone sialoprotein II (BSP II), a special odontoblast marker.^[^ ^3^, ^31^ ^]^ As shown in **Figure** **6**A,B, WB results and quantitative analyses showed that L‐Arg@ZIF‐8 failed to increase the expression of BSP II compared with the control group, while DPNMs enhanced the expression of BSP II and achieved a similar effect to iRoot BP Plus, indicating DPNMs had the ability to promote osteogenic differentiation. Furthermore, the in vivo effect was examined by immunofluorescence staining, in which BSP II and dentin matrix protein 1 (DMP1) were selected as marker proteins for the dentine layer. L‐Arg@ZIF‐8 treatment did not emit its effects on promoting osteogenic differentiation. As expected, the expression of DMP‐1 and BSP II in the DPNMs‐treated group was higher than that in the pulpitis group, suggesting that DPNMs could induce the osteogenic differentiation potential of DPSCs and promote the formation of a dentin protective layer (Figure 6C–E). The repair of the dentine layer was ascribed to the formation of HCA and inflammation alleviation. Collectively, the as‐prepared nanomotors are able to promote the osteogenic differentiation of DPSCs and repair the dentine layer.

The clinical applications of nanomaterials are often hindered by their insufficient biosafety. To explore the biosafety and systemic response, high‐dose DPNMs were injected intravenously into normal rats. The results of H&E staining suggested that the main organs including the brain, heart, liver, spleen, lung, and kidney all showed no obvious toxicity and tissue damage on both in first and seventh day post‐injection (Figure 6F). The short and long‐term results state that DPNMs have good biocompatibility, which presents great potential for clinical translation.

## 3 Conclusion

Bacterial infection and the resulting inflammatory response coupled with the destruction of the protective dentin layer are the main causes of pulpitis,^[^ ^22^, ^32^ ^]^ acting in mutual synergy and collectively leading to disease progression. These factors promote each other and together lead to the deterioration of the disease. However, current pulp capping agents cannot completely remove bacterial infections residing deep in the dental pulp tissue, and thus prevent repeated bacterial infections in the same. Moreover, the difficulty of forming a restorative dentin layer results in the continuous exposure of the pulp to the oral environment.

Nanomotors can achieve enhanced tissue penetration through self‐propelled movement in a fuel‐rich environment, however, they usually face the challenge of insufficient fuel concentration in the body. The in vivo motion performance of nanomotors can be enhanced by increasing their catalytic activity.^[^ ^13^, ^33^ ^]^ Besides, cascade reaction and dual fuel are also designed to increase fuel concentration. For instance, glucose oxidase and catalase are classic cascade systems. Glucose oxidase is used to decompose glucose to produce H_2_O_2_, supplementing endogenous H_2_O_2_. However, this system is not suitable for tissues without strong metabolism. We proposed CaO_2_ as a source of H_2_O_2_, providing new options for nanomotor design. NO production by the reaction between H_2_O_2_ and L‐Arg provided the driving force for nanomotor motion. As expected, the dual‐fuel‐propelled nanomotors exhibited superior movement performance both in vitro and in vivo. The dual‐fuel strategy will compensate for the lack of ROS in relatively mild pulpitis, expanding the application of nanomotors. Besides the nanomotors‐mediated penetration, the self‐diffusibility of NO further achieved second‐stage permeation. With the help of nanomotors, NO with antibacterial activity penetrates the bottom biofilms in the pulp cavity and perfectly eliminates bacterial infection. The two‐stage permeation strategy is crucial for deep‐seated lesion therapy.

Another important reason for selecting CaO_2_ is that the released Ca^2+^ ions facilitated dentin layer repair, induced the osteogenic differentiation of DPSCs, and thus promoted the formation of a protective dentin layer, which is important for avoiding further oral exposure. Therefore, NO‐propelled nanomotors composed of CaO_2_ and L‐Arg perfectly address the above problems that the current pulp capping agents face.

In addition, the biosafety of nanomotors always causes concerns in the clinical transformation. Our designed platform is composed of ZIF‐8, L‐Arg, and CaO_2_ nanoparticles, all of which exhibited ignorable cytotoxicity. The biodegradability and biocompatibility of these three components have been reported in a related biomedical study.^[^ ^34^ ^]^ Therefore, the developed nanomotors are expected to fulfill their therapeutic mission in the clinical treatment of pulpitis.

In conclusion, we construct dual‐fuel propelled nanomotors based on CaO_2_ nanoparticles and L‐Arg, which could respond to ROS and H^+^‐rich microenvironment to achieve enhanced motion. The two‐stage permeation strategy helps eliminate deep‐seated bacterial infections from the pulp cavity. The developed platform can be extended to other bacterial infection‐induced diseases, providing new approaches for deep infection therapy.

## 4 Experimental Section

### Synthesis of CaO_2_ Nanoparticles

First, 1 mL of CaCl_2_ (1.3 m) was added to 60 mL of anhydrous methanol under vigorous stirring (800 rpm). After 10 min, 2 mL of 3% H_2_O_2_ was slowly added. Next, 0.6 mL ammonia solution was added and kept stirring for 2 h. Finally, the solution was centrifuged at 15 000 rpm and washed thrice to obtain CaO_2_ nanoparticles.

Synthesis of DPNMs

First, 500 µL CaO_2_ (in methanol) was added into 15 mL of Zn(NO_3_)_2_•6H_2_O (20 mm) methanol solution and stirred for 30 min. Then, 15 mL 2‐methylimidazole methanol solution (40 mm) was rapidly added and stirred for 1 h to obtain CaO_2_@ZIF‐8. After that, 1 mL of 100 mg mL^−1^ L‐Arg was added into the above solution and stirred for another 4 h. Finally, DPNMs were obtained via centrifugation at 5000 rpm for 10 min and thrice washed. CaO_2_@ZIF‐8 nanoparticles were prepared according to the above steps. The difference is that L‐Arg was not added. The preparation of L‐Arg@ZIF‐8 nanoparticles also followed the above steps, with the difference being that no CaO_2_ was added.

### L‐Arg Concentration Detection

The L‐Arg gradient standard solution was added to the indicator solution which is composed of 1 mL of sodium hydroxide (1.0 m), 1 mL of 1‐Naphthol/propanol (0.6 m), and 1 mL of diacetyl/propanol (0.5 mL L^−1^) to react for 15 min. Then, the standard curve was built by measuring absorbance at 540 nm. DPNMs were incubated with 1 mm H_2_O_2_ at different times. Finally, the residual L‐Arg was tested according to the above steps. The concentration of H_2_O_2_ was determined according to the reports.^[^ ^35^ ^]^

### H_2_O_2_ Detection

DPNMs were incubated in PBS buffer (pH 6.5) at different times. Considering that ε_240_ of H_2_O_2_ was 39.4 m ^−1^ cm^−1^, therefore, the content of produced H_2_O_2_ was quantified by measuring absorbance change at 240 nm.

### NO Detection

The concentration of NO was tested by NO content assay kit based on nitrate reductase. DPNMs were incubated with 1 mm H_2_O_2_ solution or PBS buffer (pH 6.5). The supernatant containing released NO was then incubated with detection reagent for 10 min. The concentration was determined by absorbance at 540 nm and standard curve.

### Motion Behavior Measurement

1 mL of Cy5‐labeled DPNMs was added into diluted H_2_O_2_ solution and then dropped into the confocal dish. The real‐time movement behavior of DPNMs was monitored through a confocal laser scanning microscope. The motion trail was extracted from the recorded movie by ImageJ software. The self‐propelled movement behavior of DPNMs over a long time was investigated in a special device that is a two‐cell petri dish with a hole channel in the middle. Briefly, the dish was filled with PBS buffer with H_2_O_2_ (500 µm) or without H_2_O_2_. Then, 100 µL DPNMs solution (2 µg µL^−1^) was added from one side. After 30 min, 1 mL liquid was obtained from the other side and applied for ICP‐OES testing. To assess the effect of CaO_2_, the dish was filled with PBS buffer at pH 6.5. DPNMs or L‐Arg@ZIF‐8 nanoparticles were added to the dish. The other process is the same with the above steps.

### Evaluation of HCA Formation

First, 40 mg of DPNMs was weighed and dried by freeze‐drying. Next, the DPNMs were pressed into 3 mm thin circular plates using a hydraulic jack. The DPNMs‐based small discs were placed into a tube containing 20 mL of simulated body fluid (SBF) and shook (60 rpm) for 3 days. Finally, that was taken out and dried for SEM imaging.

### Antibacterial Activity Against *S. aureus* and *E. coli*

*S. aureus* was cultured in an LB medium and harvested by centrifugation. Then, bacteria were diluted to 10^4^ CFU mL^−1^ and incubated with different doses of DPNMs (0, 100, 200, 500, 1000 µg mL^−1^) at 37 °C for 0.5 h. The suspension was evenly inoculated on an LB plate, and kept in an incubator for 24 h. The CFU was counted to evaluate the antibacterial activity of as‐prepared DPNMs. Similarly, the survival rate of *E. coli* was tested by the same method.

### Human Sample

The study (no. PH2022‐B‐014) was approved by the Scientific Research Ethical Committee of Tianjin Stomatological Hospital, and written informed consents were obtained from donors. The bacteria from pulp tissue were acquired from three volunteers (volunteer 1, age 40, male, Asian; volunteer 2, age 45, female, Asian; volunteer 3, age 15, male, Asian).

### Antibacterial Activity Against Mixed Bacteria

The mixed bacteria from pulpitis patients were donated by Tianjin Stomatological Hospital. First, the obtained bacteria were cultured overnight in a BHI medium at 37 °C with continuous shaking (160 rpm). After centrifugation and collection, the bacteria were diluted to 10^4^ CFU mL^−1^ and divided into a control group, a DPNMs‐treated group, and an iRoot BP Plus‐treated group. 200 µg mL^−1^ DPNMs or the leaching solution of iRoot BP Plus was incubated with the diluted bacteria at 37 °C for 0.5 h. Then, the suspension was evenly incubated on the BHI plate and kept at 37 °C for 24 h. Finally, the CFU was counted to evaluate the antibacterial activity of DPNMs.

### Evaluation of Biomass of Biofilms

First, the biofilms were constructed by mixed bacteria collected from pulpitis patients. Briefly, 100 µL mixed bacteria in BHI medium (1 × 10^8^ CFU mL^−1^) were added to 96 well plates and then cultured in an incubator for 48 h. The culture medium was changed fresh every 24 h. The biofilms were obtained by removing the culture medium and unattached bacteria with sterile PBS. The biofilms were incubated with PBS, DPNMs, and the leaching solution of iRoot BP Plus at 37 °C for 2 h. After that, the treated biofilms were stained by 0.1% crystal violet for 10 min. Finally, the biomass of biofilms was determined via the absorbance at 595 nm and standard curve.

### Evaluation of Leaking Protein

The leaking protein was detected by the BCA protein assay kit based on bicinchoninic acid. The treatment process was similar to the above biomass evaluation. After incubation with PBS, DPNMs, and the leaching solution of iRoot BP Plus, the supernatant was added to 200 µL working solution and measured at 562 nm by UV–vis spectrophotometer. The quantification of protein leakage was determined by a standard curve.

### Animals Experiments

All animals used in this study were 220 g male Sprague–Dawley rats purchased from Beijing Vital River Laboratory Animal Technology Co. Ltd. All the animal experiments were conducted according to the Regulations for the Administration of Affairs Concerning Experimental Animals (Tianjin, revised in June 2004), and the Animal Ethics Committee of Tianjin Stomatological Hospital (approval number: PA2022‐B‐015).

### In Vivo Penetration of DPNMs

In order to evaluate whether DPNMs achieve deep penetration of dental pulp tissue, we evaluated the in vivo self‐propelled motion performance of DPNMs in pulpitis rats using IVIS imaging of Cy5 labeled ZIF‐8 or DPNMs. First, the pulp of the first mandibular incisor of SD rats was opened, and then, 20 µL PBS, DPNMs (2 µg µL^−1^), or ZIF‐8 (same concentration) were injected into the pulp cavity five times. To achieve full penetration, the pulp cavity was kept for 5 min and then sealed with dental cement at the opening. After 2 h, the distribution of nanoparticles was analyzed by IVIS III System (The excitation wavelength is 640 nm, and the emission wavelength is 680 nm).

### Penetration of DPNMs into Biofilms

According to the above method, the biofilms were constructed in a confocal dish by mixed bacteria collected from pulpitis patients. After 2 days, the biofilms were washed with PBS and stained by fluorophores SYTO 9 (6 µm) for 20 min. Next, 20 µL Cy5‐labelled DPNMs (2 µg µL^−1^) or ZIF‐8 (same concentration) was added into the dish. The penetration behavior was observed by confocal laser scanning fluorescence microscope at 3 and 10 min.

### Evaluation of Live/Dead Bacterial Viability

The abovementioned mixed bacterial biofilm was incubated with PBS, 200 µg mL^−1^ DPNMs, CaO_2_@ZIF‐8 (same concentration), or the leaching solution of iRoot BP Plus at 37 °C for 2 h. The unattached bacteria were removed by sterile PBS. Then, the biofilms were stained by fluorophores SYTO 9 and propidium iodide (PI) for 20 min. After that, the dye was gently removed by washing with PBS. Finally, the treated biofilm was collected and observed by Leica 800 confocal laser scanning microscope (Leica Microsystems, Germany), and Z was stacked into 3D images. Live bacteria showed green fluorescence, while damaged bacteria mainly exhibited red fluorescence.

### In Vivo Antibacterial Activity of DPNMs

To evaluate the antibacterial effect of DPNMs on pulpitis, a plate count test was conducted. 28 days post‐model establishment and administration, the bacteria were extracted from the pulp cavity of the pulpitis rat model. In detail, the rats were first anesthetized and perfused with physiological saline (0.9% NaCl solution). Next, the first mandibular incisor was taken, and the infected site in the pulp cavity was wiped with a swab brush for 10 s. The bacteria in the pulp cavity were taken and stored in a sterile test tube, then incubated in a culture medium at 37 °C for 24 h. After that, the bacterial suspension was diluted and cultured on the BHI agar plate for 12 h. Finally, bacterial colonies of every group were counted.

### H&E Staining

The tooth was separated at 28 days post‐injury, fixed using 4% paraformaldehyde (PFA), and then decalcified by EDTA decalcified solution. The tooth was embedded in wax for slicing. Then, the slice was successively stained by H&E. Finally, the H&E‐stained slices were imaged by an upright fluorescence microscope.

### Immunofluorescence Staining

The paraffin slices of teeth were permeabilized with 0.1% triton, blocked with normal goat serum, and then incubated with anti‐DMP1 (Santa Cruz biotechnology, #sc‐73633), and anti‐BSP II (Santa cruz biotechnology, #sc‐73630) primary antibody for 1 h. After washed thrice with TBST, the slices were incubated with FICT or FRITC conjugated goat secondary antibody for 1 h, respectively. Finally, the slices were washed thrice with TBST, stained with DAPI before sealing the surface, and observed on the upright fluorescence microscope.

### Statistical analysis

Results were analyzed by using GraphPad Prism 7 software. Differences between the two groups were assessed using unpaired *t‐tests*. For multiple comparisons, statistical significance was analyzed using one‐way analysis of variance (ANOVA), followed by Sidak's post‐hoc test, which was used when comparing all the conditions. The level of statistical significance was set at *p* < 0.05. *p < 0.05 was considered significant, and **p < 0.01, ***p < 0.001, ****p < 0.0001 were considered highly significant. All data were expressed as mean ± standard deviation (SD) unless otherwise indicated.

## Conflict of Interest

The authors declare no conflict of interest.

## Supporting information

Supporting Information

Supplemental Movie 1

Supplemental Movie 2

## Data Availability

The data that support the findings of this study are available from the corresponding author upon reasonable request.
