# Supplementary material for: Dual‐Fuel Propelled Nanomotors with Two‐Stage Permeation for Deep Bacterial Infection in the Treatment of Pulpitis
Source: Adv Sci (Weinh). 2023 Dec 3;11(5):2305063. doi: 10.1002/advs.202305063 (PMC10837366; doi:10.1002/advs.202305063)
Supplement: Supplementary file 1 — Supporting Information [file ADVS-11-2305063-s001.pdf]

ADVANCED SCIENCE
Open Access

# Supporting Information

for *Adv. Sci.*, DOI 10.1002/advs.202305063

Dual-Fuel Propelled Nanomotors with Two-Stage Permeation for Deep Bacterial Infection in the Treatment of Pulpitis

*Heping Wang, Xi Chen, Lulu Zhang, Ziwei Han, Jinxin Zheng, Yilin Qi, Weitao Zhao, Xihan Xu, Tianqi Li, Yutong Zhou, Pingping Bao* and Xue Xue**

# Supporting Information

**Dual-fuel Propelled Nanomotors with Two-stage Permeation for Deep Bacterial Infection in the Treatment of Pulpitis**

*Heping Wang, Xi Chen, Lulu Zhang, Ziwei Han, Jinxin Zheng, Yilin Qi, Weitao Zhao, Xihan Xu, Tianqi Li, Yutong Zhou, Pingping Bao*, Xue Xue**

H. Wang, X. Chen, Z. Han, Y. Qi; W. Zhao, X. Xu, T. Li, Y. Zhou, Prof. X. Xue

State Key Laboratory of Medicinal Chemical Biology, College of Pharmacy, Nankai University, Haihe Education Park, 38 Tongyan Road, Tianjin 300353, P. R. China

Email: xuexue@nankai.edu.cn

L. Zhang, J. Zheng, P. Bao

Tianjin Key Laboratory of Oral and Maxillofacial Function Reconstruction, Tianjin Stomatological Hospital, The Affiliated Stomatological Hospital of Nankai University, Tianjin 300041, China .

E-mail: pingpingbao0226@163.com

H. Wang

Current address: Key Laboratory of Radiopharmacokinetics for Innovative Drugs, Chinese Academy of Medical Sciences, Tianjin Key Laboratory of Radiation Medicine and Molecular Nuclear Medicine, Institute of Radiation Medicine, Chinese Academy of Medical Sciences & Peking Union Medical College, Tianjin 300192, P. R. China.

L. Zhang

School of Medicine, Nankai University, Tianjin 300071, P. R. China

Heping Wang,  Xi Chen contribute equally to this work

## MATERIALS AND METHODS

### Reagents and materials

Glucose was purchased from Yuanye Bio-Technology Co., Ltd. (Shanghai, China). HCl, NaCl was obtained from Bohua Chemical Regents Co., Ltd. (Tianjin, China). Ammonia, $Zn(NO_3)_2\cdot 6H_2O$ and crystal violet were purchased from Aladin Ltd. (Shanghai, China). 2-Methylimidazole and $H_2O_2$ were obtained from innochem Technology Co., Ltd (Beijing, China). Cy5 was purchased from Meilun Biotechnology Co., Ltd. (Dalian, China). L-arginine, $CaCl_2$ were obtained from Bidepharm Technology Co., Ltd (Shanghai, China). NO content assay kit was purchased from Nanjing Jiancheng Bioengineering Institute (Nanjing, China). BCA protein assay kit was purchased from Beyotime Institute of Biotechnology (Haimen, Jiangsu, China). Live/dead cell viability assay kit was obtained from Maokang Biotechnology Co., Ltd. (Shanghai, China).Polycarboxylate cement was obtained from Shofu Dental Equipment Trade Co., Ltd. (Shanghai, China). All the other reagents were obtained from Tianjin Xiangjunyunlong biotechnology Co. Ltd and all solutions were prepared by deionized water.

### Instrumentation

SEM images were obtained on a JSM-7800F SEM (JEOL Company Ltd., Japan) operated at an accelerating voltage of 30 kV. TEM images and element mapping were obtained on a JEM 2800 TEM (JEOL Company Ltd., Japan) operated at an accelerating voltage of 200 kV. FTIR spectra were recorded on a Bruker TENSOR 37 FTIR analyzer (Bruker, Germany). UV-vis spectra were determined by a Shimadzu 2550 UV-vis scanning spectrophotometer (Shimadzu, Japan). The absorbance was detected using the microplate reader (Tecan, Austria). The fluorescent images were observed by V5800 inverted fluorescence microscope (Viyee, China) and LSM 800 with Airyscan confocal laser scanning fluorescence microscope (Zeiss, Germany). The fluorescent images of mice were captured by IVIS spectrum (Caliper, USA). The particle size was determined by dynamic light scattering (Malvern, United Kingdom).

### Measurements of loading content and encapsulation efficacy

The amount of L-Arg was determined by spectrophotometry. DPNMs were rapidly solubilized in an acidic solution and then added to the indicator solution which composed of 1 mL of sodium hydroxide (1.0 M), 1 mL of 1-Naphthol/propanol (0.6 M) and 1 mL of diacetyl/propanol (0.5 mL/L) to react for 15 min. Then, the concentration was determined through measuring absorbance at 540 nm and referring to standard curve. The amount of $CaO_2$ was detected by ICP-OES.

The loading content was calculated by the following equation (1).

$$\text{Loading content (\%)}=\frac{\text{weight of drug in DPNMs}}{\text{weight of DPNMs}}\times 100$$

The encapsulation efficiency was calculated by the following equation (2).

$$\text{Encapsulation efficiency (\%)}=\frac{\text{weight of drug in DPNMs}}{\text{weight of total drug added}}\times 100$$

**Analysis of the proinflammatory cytokines**

The pulp was separated and homogenized in 1 mL Trizol at 28 d post injury. RNA was transferred to the aqueous phase through chloroform, precipitated by isopropanol, and finally separated by centrifugation. The obtained RNA was quantified by NanoDrop 8000. A certain amount of RNA was used to invert into cDNA and further quantified by quantitative real-time polymerase chain reaction (qPCR). β-actin functioned as endogenous housekeeping gene to normalize the corresponding mRNA. The mRNA expression level was calculated based on comparative Ct method ($2^{-\Delta\Delta Ct}$). All of primers were designed by Primer-BLAST (National Center for Biotechnology Information) and listed in **Table S1**.

**Western blotting analysis**

Western blotting analysis was performed to detect protein expression of representative proinflammatory factors (TNFα) and bone sialoprotein II (BSP II). Total protein lysate from pulp was prepared by RIPA lysis buffer (BOSTER, China), sonicated on ice, and centrifuged at 13400 *g* at 4 °C. The protein concentrations were determined using a BCA protein assay kit and analyzed with SDS-polyacrylamide gel electrophoresis and subsequently transferred to a polyvinylidene difluoride membrane. The anti-TNF-α (1:1000, #ab255275), anti-BSP II (1:1000; #sc-73630), and anti-β-Tubulin (1:1000; #BE0025) antibodies were purchased from Abcam, Santa cruz biotechnology and Easybio, respectively. The horseradish peroxidase (HRP)-conjugated secondary antibody was purchased from Abcam. Densitometric scanning normalized against β-Tubulin was used to assess relative protein concentration. Blots were imaged by imaging system (Tanon5200, Shanghai, China).

***In vivo* biosafety analysis**

The *in vivo* toxicity of DPNMs was assessed by H&E staining. 100 μL DPNMs (2 mg/mL) were intravenously injected into mice. The main organs including brain, heart, liver, spleen, lung, kidney were separated at 1 d and 7 d post injection, fixed using 4% PFA, and then embedded for frozen section. The H&E-stained slices were imaged by an upright fluorescence microscope. 100 μL PBS was as control.

**Statistical analysis**

Results were analyzed by using GraphPad Prism 7 software. Differences between two groups were assessed using unpaired *t* tests. For multiple comparisons, statistical significance was analyzed using one-way analysis of variance (ANOVA), followed by Sidak's post-hoc test, which was used when comparing all the conditions. The level of statistical significance was set at $p < 0.05$. *$p < 0.05$ was considered significant, and **$p < 0.01$, ***$p < 0.001$, ****$p < 0.0001$ were considered highly significant. All data were expressed as mean ± standard deviation (SD) unless otherwise indicated.

**Supplementary Figures**

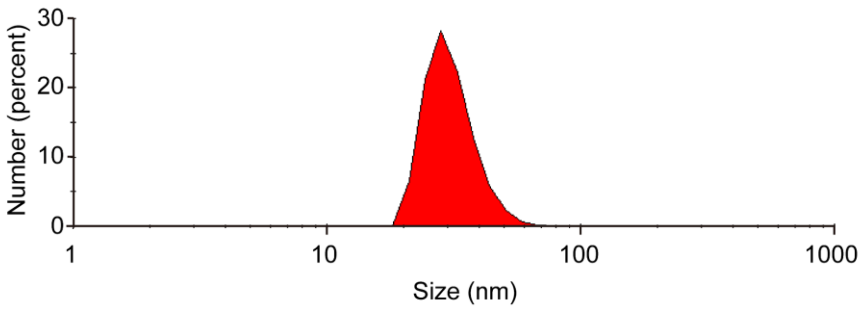


**Figure S1.** Size distribution of $CaO_2$ nanoparticles by dynamic light scattering (DLS).

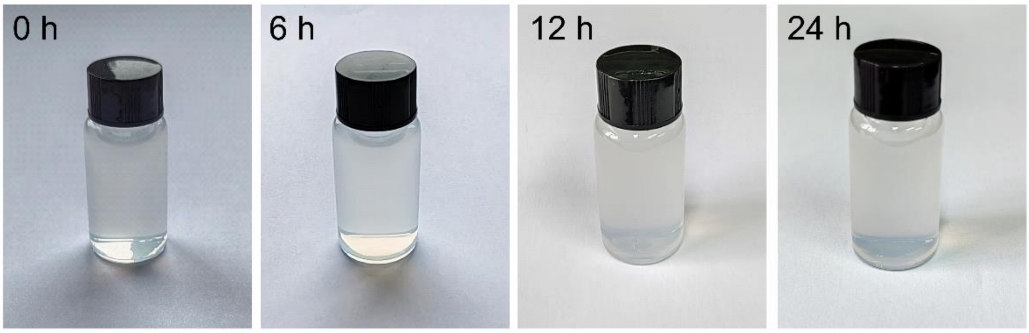


**Figure S2**. The photographs of DPNMs dispersed in aqueous solution at different time points.

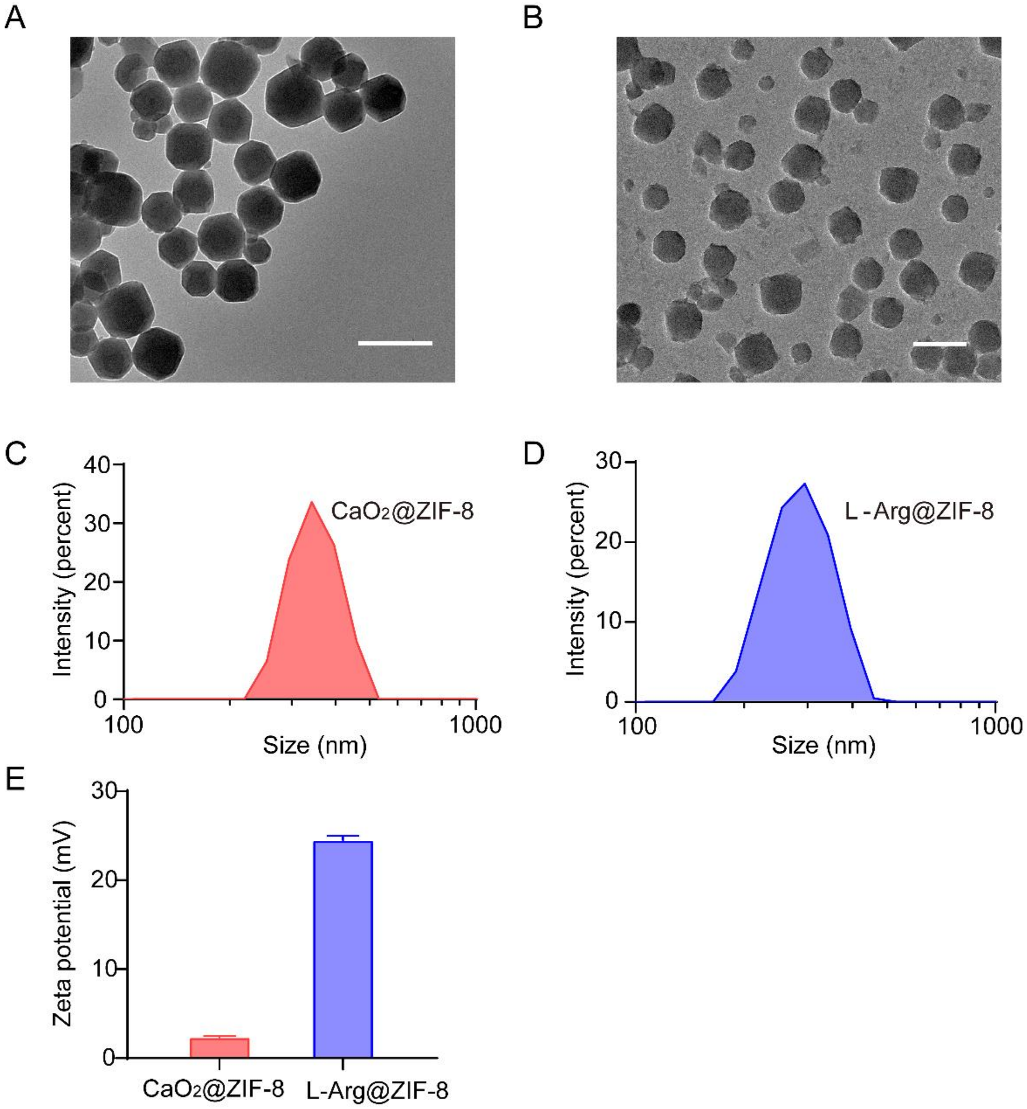


**Figure S3**. (A, B) TEM images of (A) $CaO_2$@ZIF-8 nanoparticles and (B) L-Arg@ZIF-8 nanoparticles. Scale bar, 500 nm. (C, D) Size distribution of (C) $CaO_2$@ZIF-8 nanoparticles and (D) L-Arg@ZIF-8 nanoparticles. (E) Zeta potential of $CaO_2$@ZIF-8 and L-Arg@ZIF-8 nanoparticles.

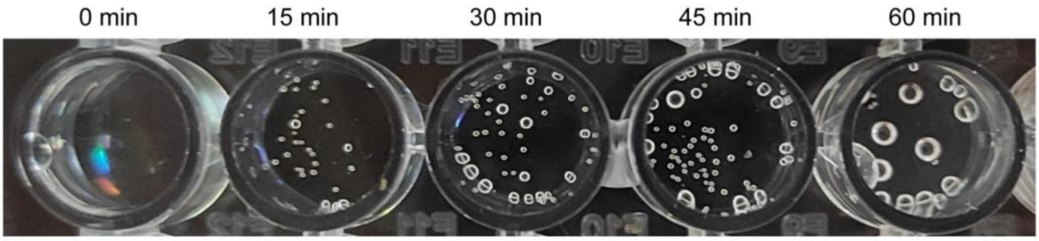


**Figure S4.** Photograph of formed NO bubbles at predetermined time points.

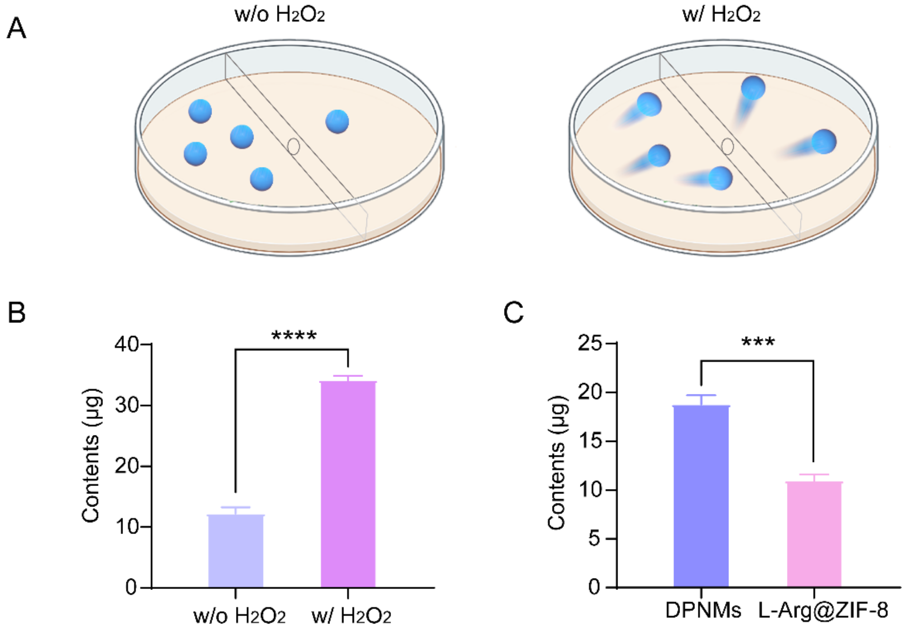


**Figure S5**. (A) Scheme illustration of the special device and movement behavior with/without $H_2O_2$. (B) The penetrated content of DPNMs in the solution with/without $H_2O_2$. (C) The penetrated content of DPNMs or L-Arg@ZIF-8 in solution at pH 6.5. Data are presented as mean ± SD (n = 3, unpaired Student's *t*-test, ***$p < 0.001$, ****$p < 0.0001$).

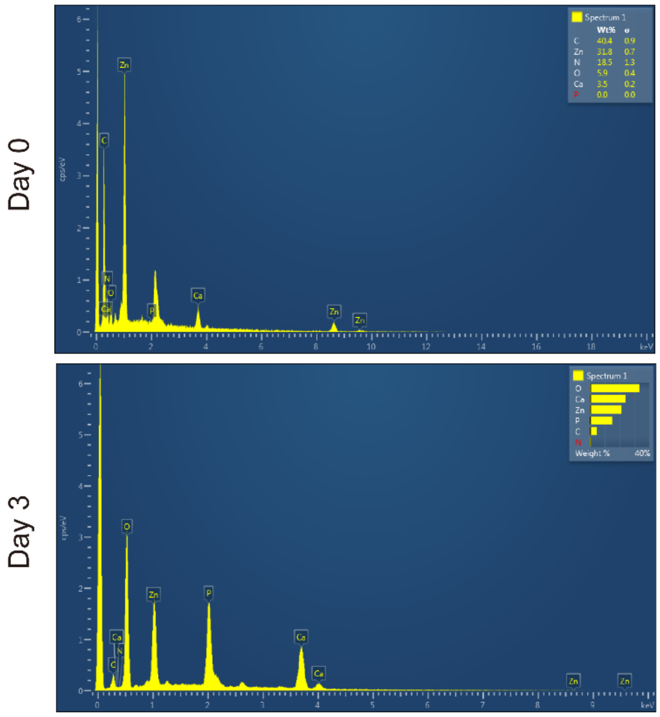


**Figure S6**. The element analysis of DPNMs before and after immersion in SBF solution for 3 days.

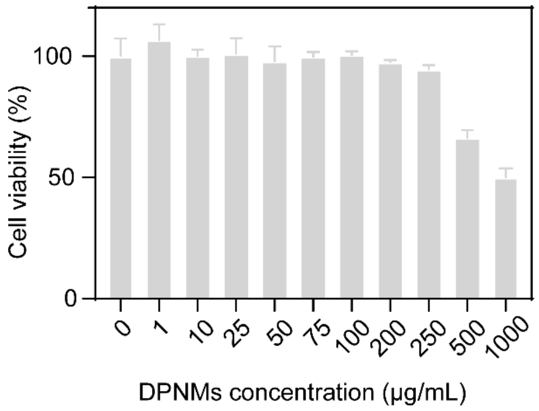


**Figure S7**. The DPSCs cells viability under different concentrations of DPNMs by 3-(4,5)-dimethylthiahiazo(-z-y1)-3,5-di-phenytetrazoliumromide (MTT) assay. Data are expressed as mean ± SD (n = 3).

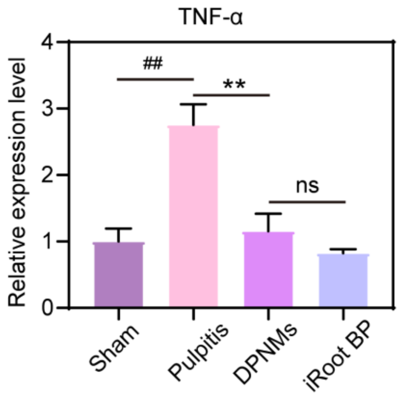


**Figure S8**. The quantization of TNF-α expression in Figure 5C. Data are expressed as mean ± SD (n = 3, one-way ANOVA and Sidak's multiple comparison tests, $^{\#\#}p < 0.01$ versus sham group, and $^{**}p < 0.01$ versus pulpitis group, ns means no significance).

**Table S1**. qPCR primer sequences.

**Table S1**. qPCR primer sequences.

| *gene* | Forward (5'-3) | Reverse (5'-3) |
|---|---|---|
| *il1β* | TGTGTAATGAAAGACGGCA | TCCACTTTGCTCTTGACGGCAC |
| *tnfα* | CAAAATTCGAGTGACAAGCCT | CTGGGAGTAGACAAGGTACAAC |
| *β-actin* | GGCTGTATTCCCCTCCATCG | CCAGTTGGTAACAATGCCATGT |
